# Building leadership capacity to prevent and control noncommunicable diseases: evaluation of an international short-term training program for program managers from low- and middle-income countries

**DOI:** 10.1007/s00038-016-0913-x

**Published:** 2016-12-09

**Authors:** Agnes Erzse, Pascal Bovet, Fred Paccaud, Oleg Chestnov, Nicholas Banatvala

**Affiliations:** 1 World Health Organization, Noncommunicable Diseases and Mental Health Cluster, Geneva, Switzerland; 2 University Institute of Social and Preventive Medicine, Route de la Corniche 10, 1010 Lausanne, Switzerland

**Keywords:** Noncommunicable diseases, Training, Career trajectory, Low- and middle-income countries

## Abstract

**Objectives:**

To assess the impact of a 1-week training seminar jointly developed and conducted by the World Health Organization and the University Institute of Social and Preventive Medicine of Lausanne targeting senior policy-makers in low- and middle-income countries on public health aspects of noncommunicable diseases (NCDs).

**Methods:**

A short qualitative questionnaire was emailed to all participants to one of the nine seminars organized between 2010 and 2015.

**Results:**

From the 195 participants from 96 different countries, 122 (63%) completed the questionnaire. Among them, 87% reported that the seminar made a positive contribution to their professional development and 48% said it helped strengthening their national NCD program. All respondents remained directly or indirectly involved in NCD work. A frequent suggestion was that similar seminars are developed in their region or country.

**Conclusions:**

The evaluation strongly suggests that this short-term seminar had positive impact on both participants’ personal development and the organization they worked for. There is a demand for organizing similar seminars at regional/country levels to support NCD prevention and control programs.

## Introduction

Noncommunicable diseases (NCDs), mainly cancer, diabetes, cardiovascular disease, and chronic respiratory disease, have become critical health and development challenges in low- and middle-income countries (LMICs) (GBD collaborators 2015a). These NCDs cause significant preventable premature mortality and share several major risk factors, particularly tobacco use, harmful use of alcohol, unhealthy diet, and physical inactivity (GBD collaborators 2015b). Interventions to reduce the NCD burden, including in LMICs, have been formulated and the response largely relies on effective public health interventions (World Economic Forum 2011; Bonita et al. 2013; WHO 2013). The roots and determinants of NCDs extend largely beyond the health sector, which underlies the need for capacity building programs that address multisectoral action, including the need for “health in all policies”, “whole-of-government” and “whole-of-society” approaches (WHO 2012; Greenberg et al. 2011; Siegel et al. 2011).

This emphasizes the importance to include public health aspects of NCDs in training programs for public health professionals (Greenberg et al. 2015). However, policy-makers, program managers, and health professionals often lack optimal knowledge and skills to effectively prevent and control NCDs and capacity building programs in this area are scarce in LMICs. Indeed, most public health training programs in LMICs have not yet reoriented their curriculum to include NCD prevention and control (Siegel et al. 2011) and the focus is often limited to clinical care rather than public health interventions where this has been done (Greenberg et al. 2015; Thakur et al. 2011; Schmidt et al. 2015).

In response to this challenge, the World Health Organization (WHO) and the University Institute of Social and Preventive Medicine in Lausanne (IUMSP) established in 2010 a training program called “Seminar on the Public Health Aspects of NCDs” to target senior officers from LMICs involved in national NCD prevention and control programs. The structure of the seminars was also built on IUMSP’s previous experience in delivering training programs on public health aspects of NCDs conducted in several LMICs (Bovet et al. 2003). The seminars took place in Lausanne with one day spent at the WHO headquarters in Geneva. By 2015, 9 seminars had been held and 195 participants had participated from 96 countries from all 6 WHO regions.

The main participants are senior policy-makers in charge of national or subnational NCD prevention programs in ministries of health. Other participants include health professionals working in national WHO offices, public health organizations, academic institutions, and public health agencies, who are collaborating with ministries of health. The seminars were structured around the leadership and public health skills required to deliver the WHO Global NCD Action Plan.

The content of the seminars is based on a core set of presentations from a faculty consisting of a number of senior staff from WHO, universities, and other agencies, as well as from the participants themselves. Emphasis is given to facilitated group discussions and group work. Principles for prevention and control of NCDs, surveillance, multisectoral action, leadership, partnership, advocacy, and health system strengthening are all core parts of the training program. Emphasis is given to the WHO Global Action Plan of NCD prevention and control (WHO 2013) and other related documents. Experiences and lessons from participating countries are explored as well as avenues for strengthening prevention and control programs in participants’ countries. A website (www.ncdseminar.org) includes materials used during the seminars and a group on Facebook has been developed to facilitate interaction between current and past participants.

In 2015, we undertook an evaluation to: (1) assess the impact of the seminars at the participants and their organizations levels; (2) track the professional development trajectory of the participants; and (3) gain feedback from the participants for the benefit of future seminars.

## Methods

The evaluation took place between July and August 2015. The authors emailed the questionnaire to each participant who attended one of the nine seminars that took place between 2010 and 2015, with a request for the completed questionnaire to be emailed back to the authors. Participants were informed that the data would be anonymized before analysis and analysis would be carried out with aggregate data only. The questionnaire included 17 closed- and open-ended questions. Up to five reminders were sent at regular intervals over a period of 3 months to the non-respondents. Results are tabulated by regions. Chi-square test or *t* test was performed to test for differences between proportions or continuous variables, respectively, using the statistical software Stata 13.0.

The study, which retrospectively evaluates the impact of the training program on the participants, was approved by the organizing institutions, i.e., the World Health Organization, Geneva, and the University Institute of Social and Preventive Medicine, Lausanne. Participants to this study were free to participate. By completing the questionnaire sent by email, which also explained the purpose of the survey, participants agreed to participate to the study.

## Results

### Participants

From the 195 eligible participants, 122 completed the questionnaire (63%). Among non-respondents, 7 acknowledged the survey but did not complete the questionnaire and 66 did not respond to the emailed survey. Data did not allow distinguishing whether non-respondents received the survey email and did not respond or whether they did not receive the email because their email address was no longer valid.

Eligible participants came from 96 countries from all 6 WHO regions. The response rate was similar across the 6 regions (Table 1). Participation to the survey was greater among those participants who attended more recent seminars, ranging from around 45% in 2010 to around 70% in 2014 and 2015. The response rate was 16% shortly after sending the original first email, 14% upon a first reminder, 15% upon a second reminder, and 11% upon subsequent 4th and 5th reminders. There was no difference in gender, age or interest in the seminar between participants who responded before (*n* = 61) or after the deadline (*n* = 61). However, a higher proportion of late vs. early responder participants had changed their job (respectively, 32 vs. 17%, *p* = 0.012).

**Table 1:** Numbers of eligible participants and response rate by WHO region

	Eligible participants (*n*)	Completed the questionnaire (%)
AFRO	23	14 (61)
AMRO	22	14 (64)
EMRO	54	32 (59)
EURO	40	27 (67)
SEARO	33	19 (58)
WPRO	23	16 (70)
Total	195	122 (62)

*AFRO* African region, *AMRO* Americas region, *EMRO* Eastern Mediterranean region, *EURO* Europe region, *SEARO* South East Asia region, *WPRO* Western Pacific region

Of the 122 participants surveyed, 44% were from ministries of health, 22% from WHO country or regional offices, 7% from universities and 27% from other health institutions. The sex and age distribution of the 122 participants according to WHO region is shown in Table 2. There were, overall, similar proportions of male and female participants and of persons aged less or more than 45 years, with some non-significant differences according to WHO region (Chi square *p* > 0.05).

**Table 2:** Demographic distribution (in percent) of participants by WHO region

	AFRO	AMRO	EMRO	EURO	SEARO	WPRO	Total
	(*n* = 14)	(*n* = 14)	(*n* = 32)	(*n* = 27)	(*n* = 19)	(*n* = 16)	(*n* = 122)
Male	57	31	52	37	74	60	51
Female	43	69	48	63	26	40	49
Age <45	54	46	70	59	37	67	57
Age ≥45	46	54	30	41	63	33	43

*AFRO* African region, *AMRO* Americas region, *EMRO* Eastern Mediterranean region, *EURO* Europe region, *SEARO* South East Asia region, *WPRO* Western Pacific region

### Satisfaction and impact of the seminar on the participants

The average rating given by the respondents for the seminars was 8.1 on a scale of 1 (poor) to 10 (excellent). The mean level of satisfaction with the seminar did not substantially differ according to WHO region, as shown in Table 3. Eighty nine percent of the respondents said that they had referred to the seminar website at least once since returning from the seminar. Forty seven percent of the respondents reported having had discussions about NCDs with other participants after the seminar and 25% of those who have not had such follow-up discussions reported that they would like to have a discussion forum on the seminar website. Seventy-five percent of the respondents indicated that they would like to participate again in the seminar and the remaining 25% reported that they would consider participating again.

**Table 3:** Impact of the seminar on the individual participants by WHO region

	AFRO	AMRO	EMRO	EURO	SEARO	WPRO	Total
Referred to seminar’s materials ≥4 times (*n* = 118) (%)	93	69	94	85	82	93	87
Had follow up discussion with other participants (*n* = 113) (%)	5	38	50	38	56	43	47
Willing to participate again (*n* = 114) (%)	92	85	75	76	65	64	75
Rating of course (range 0–10)	8.1	7.8	8.2	8.3	8.0	8.1	8.1

Data are presented as percentage except for rating of the course

*AFRO* African region, *AMRO* Americas region, *EMRO* Eastern Mediterranean region, *EURO* Europe region, *SEARO* South East Asia region, *WPRO* Western Pacific region

### Application of knowledge acquired during seminars

Eighty-seven percent of the respondents said that they had applied knowledge they had acquired during the seminars “a lot”, and 92% said that the benefit of the seminars lasted “12 months or more” (Table 4). When describing how they used the knowledge acquired, the most common response was in terms of helping them develop national strategic and policy documents for NCDs within their organization.

**Table 4:** Impact of the seminar at the individual and organizational levels (in percent) by WHO region

	AFRO	AMRO	EMRO	EURO	SEARO	WPRO	Total
Applied the seminar knowledge “a lot” vs. “a little” (*n* = 118)	92	69	94	85	82	193	87
Benefits of the seminar lasted for more than 12 months vs. less (*n* = 118)	100	100	100	96	82	100	97
The organization that I work for supported me in implementing what I learned during the NCD seminar(s) (*n* = 116)	64	46	72	73	57	71	66
After the NCD seminar(s) I had discussions with my colleagues about what I learned and how to apply the knowledge and skills I gained (*n* = 118)	100	77	97	100	88	100	95
My colleagues were open to changing the way things are done in relation to NCD program and policy (*n* = 115)	93	83	88	88	81	93	88
My supervisor discussed with me on how to apply what I learned at the NCD seminars in relation to NCD program and policy (*n* = 115)	86	62	78	88	69	79	78
My supervisor was open to changing the way things are done in relation to NCD program and policy (*n* = 113)	100	83	84	92	73	93	88

*AFRO* African region, *AMRO* Americas region, *EMRO* Eastern Mediterranean region, *EURO* Europe region, *SEARO* South East Asia region, *WPRO* Western Pacific region

With the help of the seminar knowledge, I provided technical assistance in the development of the National NCD Strategy and in the development of the regulations on the reduction of salt in selected foods.

Respondents also highlighted the benefit from the technical guidance provided during the seminars, for example in relation to the WHO STEP surveys and other NCD surveillance tools, the WHO Package of Essential Noncommunicable (PEN) Disease Interventions for Primary Health Care in Low-Resource Settings, and the most cost-effective and feasible interventions (“best buys”) to prevent and control NCDs that are described in the WHO Global Action Plan 2013–2020.

I applied the tobacco control best buy, to advocate for passage of the national tobacco control bill, which has now been passed into law.

A large number of respondents said that they had been encouraged by the seminars to engage more in political advocacy of NCD-related issues, to accelerate efforts in establishing an NCD program unit, and to work toward the prioritization of NCDs in their national health action plans. Furthermore, respondents applied the new skills and knowledge in their everyday lives in preparation for meetings, presentations, and lectures for university students.

I used the seminar knowledge on many occasions I had to discuss with high-level decision makers.

Responses of participants also revealed a shift in thinking from models of health care to models based on public health with action on broader social determinants of health.

Developing better knowledge on the broader determinants of NCDs helped me consider not only treating patients but also focusing on different sectors of the society to prevent NCDs in the population.

### The impact of the seminar at the organization’s level

Participants were asked to what extent their organizations support them in the application of their new knowledge and skills acquired during the seminar (Table 4). Organizations were reported to be relatively open to the knowledge gained from the seminar and participants reported having often been asked to share their experiences with their colleagues, other health professionals and representatives from different sectors.

I advocated and assisted the National Institute of Public Health to integrate NCD training in the Master of Public Health curriculum.

A large proportion of respondents reported that their organization was open to benefit from the participants’ improved understanding on NCD prevention and control, and updated advocacy and leadership skills.

My organization is better focused to provide guidance to countries on NCDs and the Global Action Plan due to my new knowledge and as I heard of other countries’ experiences.

The knowledge learnt at the NCD seminar helps my organization in how to best design laws, projects, and programs for the prevention of NCDs.

The application of the new knowledge also offered expanded contribution to the policy arena; encouraged organizations to advocate the prioritization of NCDs; and helped allocate a larger share of the budget for NCD prevention and control strategies.

The seminar helped me in putting NCD prevention and control as a priority among all national health projects.

The seminar helped me to accelerate advocacy and leadership skills for NCD prevention and control.

### Participants’ professional development

All respondents said that they were still working in the area of NCDs, 70% directly (e.g. as a national advisor of NCDs) and 30% indirectly (e.g., as a specialist in public health in a department/unit of the health system).

Nevertheless, 42% of respondents had changed their jobs since attending the seminar with 78% identifying this change as a promotion (Table 5). Table 5 also shows that 82% of respondents had engaged in professional development activities related to NCDs since the seminar. The most frequent being workshops addressing the global burden of NCDs; disease-specific seminars for health professionals; high-level expert meetings and consultations on the development of NCD strategies and policies; and training programs which improve management skills of health workers for more effective and efficient management of programs and projects.

**Table 5:** Individual career trajectory (in percent) by WHO region

	AFRO	AMRO	EMRO	EURO	SEARO	WPRO	Total
Position directly linked to NCDs at the time of the seminar(s)	86	86	72	67	68	50	70
Position indirectly linked to NCDs at the time of the seminar(s)	14	14	28	33	32	50	30
Current position directly linked to NCDs	86	62	61	56	47	63	61
Current position indirectly linked to NCDs	14	38	39	44	53	38	39
New position since the seminar	38	62	52	37	32	27	42
Job change was a promotion	60	100	88	80	50	50	78
Further development of activities in the area of NCDs	82	69	80	92	68	100	82

*AFRO* African region, *AMRO* Americas region, *EMRO* Eastern Mediterranean region, *EURO* Europe region, *SEARO* South East Asia region, *WPRO* Western Pacific region

## Discussion

This evaluation suggests that the seminar had a positive impact from both the respondents’ personal perspective and for the organizations in which they worked. The seminar was able to raise participants’ aspirations, motivation, and attitudes to respond to the challenge of NCDs. The data also demonstrate that respondents remained working in the area of NCDs and that a significant number had been promoted.

From the perspective of the impact of the seminar on the organizations in which participants worked, many participants reported a positive response on their return from their managers and colleagues. It seems that the seminar was therefore able to empower the participants and improve their organizations’ ability to provide strategic leadership on NCDs. Participants often reported acting as a source of knowledge and expertise in local NCD workshops and high-level meetings, and they reported that their recommendations were increasingly being translated into action. Participants also reported being better able to take up their roles as focal points of NCD prevention and control, and were better able to provide technical assistance to the ministry.

The findings also suggest that the seminar was successful in convincing participants that NCD prevention and control should largely rely on public health interventions and that participants were adequately equipped to encourage their organizations to apply this approach more broadly and effectively. This is consistent with the modern principles of public health training that McKee has described (McKee 2012): (1) stimulate curiosity of participants; (2) encourage people to take initiatives and become social entrepreneurs; (3) encourage people to make connections and look upstream to identify the causes of the causes; (4) convey the big picture of issues; (5) clarify what people stand for; (6) support health professionals to engage with key decision makers at all levels; and (7) ensure that approaches to public health are firmly grounded in human rights.

It would be important that participants continue communicating with each other, which stresses the need for effective platforms for continued interaction and discussion. This is especially important as NCD program managers form an important network for the prevention and control of NCDs worldwide. The use of existent social media platforms such as Facebook (around 1.3 billion users), Twitter (around 300 million users) or similar social networks, which are highly organized, user-friendly, and consistently well maintained, may possibly be efficient alternatives to long-term networking as compared to dedicated NCD platforms that are demanding in maintenance, not always sufficiently user-friendly, and consequently often short-lived.

There are several limitations with this evaluation. First, the total number of participants was fairly small, which prevented detailed statistical analysis. Second, the analysis is the result of the subjective views of participants. Third, the response rate to the survey was less than 100%. Nevertheless, the response rate of 62% is higher than the 33% described in a recent review of participation rates in similar surveys (Nulty 2008). The lower completion of the survey questionnaire by participants attending earlier than later seminars may be attributable to the possibility that email addresses might have been out of date among the former. Fourth, there may have been some social desirability effect toward favorable responses as the questionnaire was not anonymous, particularly since the survey was conducted by the organizers of the seminar, but we believe that this potential bias was limited and did not significantly alter the results. Fifth, we consider it highly unlikely that the reduction in the length of seminars from 8 to 5 days over the six years impacted on the results of the evaluation. Finally, the evaluation was not able to assess the impact of the seminar on changes in NCD-related policy or specific health outcomes as this would be very difficult to assess.

Strengths of the survey include, first, that it targeted all participants from the seminars organized to date and, second, that the survey assessed the long-term impact, which is rarely assessed in evaluations of training programs since most such evaluations are conducted during or immediately after the end of training programs.

To date the seminars have targeted senior officials in ministries to build leadership among this group. As this capacity is developed, there may be opportunities to tailor seminars for senior officials from other sectors that are relevant to the prevention of NCDs, for example finance, labor, education, and trade and investment.

In summary, our data indicate that a short training program focusing on the public health aspects of NCDs targeting program managers in LMICs had a positive impact on both the professional development of the participants and capacity of their organizations. The increasing need for LMICs to respond to NCDs highlights the value of these sorts of capacity building programs and the need for more to be developed, particularly in LMICs.
